# 

**Published:** 1884-10

**Authors:** 


PAGE

ORIGINAL COMMUNICATIONS.—Homœopathy. Frank S. Billings, V.S. .................... 309
Does Scarlet Fever exist in the Lower Animals? Thomas Walley, M.R.C.V.S. .............. 326
Contagious Animal Diseases in Western New York. Edward Crundall, V.S. ................ 330
History of Tuberculosis. Dr. Albert Johne .................................... 338
The Prevention of Contagious Pleuro-pneumonia in Cattle ........................ 351
Hæmoglobinæmia Toxica .................................................. 361
EDITORIAL.—To the Profession.—The Bureau of Animal Industry—Pleuro-Pneumonia—The Bacillus of Abortion—Scarlet Fever in the Lower Animals—The Veterinary School at Milan, Italy—Annual Report of the Royal Veterinary Institute at Vienna, for the year 1883—Infectious Bronchitis Fibrinosa in Cattle—Meat Inspection at the Munich Abattoir—The Action of Different Proportions of Chemical Agents upon the Germs of Emphysema Infectiosum—Report of the Munich Veterinary Institute for the year 1883—Examination of Pork for Trichinæ at Hamburg .................................................. 376
CORRESPONDENCE .................................................. 391
CASE DEPARTMENT.—Radical Cure of Umbilical Hernia in a Colt—A Case of Enteritis ....... 392
REVIEWS.—Animal Castration—Handbuch der Vergleichenden Histologie und Physiologié der Haussangelthiere—Handbook of Comparative Histology and Physiology of the Domestic Animals—How to tell the Age of a Horse—The American Liveryman and Horse-owner—Anatomy of the Horse—Books and Pamphlets received ........................ 394
REPORTS.—Ohio State Veterinary Medical Association ............................ 397
NEWS AND MISCELLANY .................................................. 399

